# Botulinum Neurotoxins: From Toxin to Medicine

**DOI:** 10.3390/toxins15100621

**Published:** 2023-10-20

**Authors:** Andrea Santamato

**Affiliations:** Spasticity and Movement Disorder-ReSTaRt Service Unit, Physical Medicine and Rehabilitation Department, Policlinico of Foggia, University of Foggia, 71122 Foggia, Italy; andrea.santamato@unifg.it

Ancient scientific manuscripts indicate that Dr. J. Kerner was the first, between 1817 and 1822, to suggest the possible therapeutic use of botulinum toxin, which he called “sausage poison”, describing it in the final chapter of his 1822 monograph; based on his previous experiences, he concluded that the toxin, applied in minimal doses, would lower or block the hyperactivity and hyperexcitability of the overactive nervous system.

Therefore, the name botulinum is strictly related to the word botuls (from the Latin word botulus, meaning “sausage”) named in this way by the German physician Muller in 1870, whereas at the end of 1800, E.P. Van Ermengem identified a bacteria and its toxin [1,2].

It is fascinating to consider that after almost 100 years, the botulinum toxin (BoNT) has finally become an important drug for many human diseases.

It is known that BoNT is a protein consisting of a heavy chain and a light chain linked via a disulfide bond. The activity of BoNTs involves a multistep process of heavy-chain-mediated cell binding to neuronal cells, endocytosis, and translocation of the light chain into the cytosol, followed by cleavage by the light chain of soluble N-ethylmaleimide-sensitive factor attachment receptor (SNARE) proteins. The cleavage of SNARE proteins by the zinc endopeptidase action of the light chain results in the disrupted fusion of synaptic vesicles containing neurotransmitters with the plasma membrane [3].

Botulinum neurotoxin is similar to a chemical scalpel that reduces the activity of cholinergic junctions inducing, for example, reversible muscle denervation.

Its possibility to have this effect is highly appreciated in the medical field, especially for spasticity, strabismus, blepharospasm, cervical dystonia, axillary hyper-hidrosis, glabellar lines, migraine, and neurogenic bladder [4].

Many serotypes produced by clostridium botulinum have been identified (A,B,C, D,E,F, and G), many of them with different subtypes, target proteins, and cleavage sites, but only BoNT/A and BoNT/B are used as medical therapies, showing safety and efficacy.

Therefore, “From toxin to medicine” is the title of this Special Issue of *Toxins* as many years have passed, and the efficacy of this toxin for therapeutic interventions has been fully described in several published scientific studies.

It is very important that this Special Issue includes the study of Shin-Ichiro Miyashita et al, as they investigated the efficacy of BoNT/CD, a mosaic toxin of BoNT/C and BoNT/D, assessing its potential as a therapeutic alternative to BoNT/A [5].

The authors showed, in a cultured neuron assay, that BoNT/CD cleaved syntaxin and SNAP-25 more than BoNT/C and BoNT/A, and after mouse digit abduction muscle injection, BoNT/CD induced dose-dependent muscle paralysis lasting ~21 days whereas BoNT/A-induced paralysis lasting ~30 days. BoNT/C failed to induce local paralysis without systemic toxicity.

The authors concluded that BoNT/CD may be a potential alternative for patients who do not respond to existing BoNT-based therapeutics.

Until now, in the clinical setting, commercial BoNT-A products have been formulated as a powder for reconstitution before injection with saline solution.

However, new products in a ready-to-use liquid formulation are appearing on the market. Elena Fonfria et al., described, in an interesting study, the suitability of a potency method that uses the BoCell^®^ assay as a replacement for the LD50 assay to test aboBoNT-A in both powder and liquid formulations.

Following the assay validation and comparability studies presented here, the BoCell^®^ assay has received approval from regulatory authorities in the European Union (both powder and liquid formulations) and in the USA (powder formulation) for establishing the potency, batch release, and stability purposes of aboBoNT-A, whereas submissions and approvals in other geographic areas are ongoing [6].

As the title of this Special Issue is “from toxin to medicine”, this implies that a linear progression has occurred; therefore, following the studies cited before, focused on biological effects and methods to demonstrate them, clinical studies produced by clinicians with great expertise on spasticity, movement disorders, and pain have been collected.

Bensmail and colleagues described the real-world use of BoNT-A for spasticity and neurogenic detrusor overactivity treatment on 105,206 multiple sclerosis subjects identified using the electronic health records of those treated with BoNT-A injection within striated muscle for spasticity and/or within the detrusor smooth muscle for neurogenic detrusor overactivity. The authors studied different numbers of subjects who received BoNT-A injection, ranging from >1 to >3 treatment, and reported several therapeutic results depending on the number and proportion of patients who received at least one BoNT-A injection, the number and proportion of patients who received at least three BoNT-A injections, and the time interval between two BoNT-A injections administered; moreover, they examined the rate of intradetrusor BoNT-A treatment, the mean number of intradetrusor BoNT-A injections per patient, and the time interval between two intradetrusor BoNT-A injections.

This study reinforces the importance of repeated BoNT-A injections to maintain a satisfactory therapeutic outcome against spasticity and neurogenic detrusor overactivity in subjects with multiple sclerosis [7].

One of the first indications BoNT therapy was approved by the FDA for was cervical dystonia, and currently BoNT is recommended as the first-line treatment for cervical dystonia (CD), with statistically significant improvements seen in 70–90% of patients in clinical studies [8]. Accurate diagnosis, appropriate pattern classification, physician experience, and correct BoNT-A dose and precision of injections are the most important factors contributing to the successful treatment of cervical dystonia.

The ultrasound guide for injection has been shown to be quick and painless, and it provides the real-time visualization of muscles and adjacent anatomical structures in different studies, especially for spasticity treatment, compared with other injection techniques [9].

In this Special Issue, Tyślerowicz and colleagues evaluated the efficacy of ultrasound-guided BoNT-A injections in comparison with anatomic landmarks in patients with cervical dystonia, using the Toronto Western Spasmodic Torticollis Rating Scale, Tsui modified scale, Craniocervical Dystonia Questionnaire, and Clinical Global Impression—Improvement scale. The authors concluded that ultrasound guidance might be helpful in improving the results of BoNT-A injections in cervical dystonia, reducing associated pain and disability [10].

Pain is the most common non-motor symptom of cervical dystonia, affecting 55–90% of patients with CD, and is rated as moderate or severe by 71% of patients; it is often the main reason patients with CD seek treatment and is a major contributor to disability and impaired QoL [11].

The efficacy of BoNT-A in relieving pain in patients with CD has been confirmed in analyses of controlled clinical trial data and in large-scale observational studies/registries of CD patients [12,13,14].

BoNT-A has been found to inhibit bradykinins, serotonin, potassium, prostaglandin E2, substance P, and the neuropeptide CGRP, reducing the sensitization of muscle nociceptors [15]. Moreover, BoNT injections have been used to treat muscle pain evoked by chemical C and A fiber stimulation because of the local production of lactate and acid PH induced by prolonged muscle activity and long-term contracture.

The published paper into this Special Issue described a pooling data from four studies in cervical dystonia adults treated with BoNT-A (incobotulinumtoxinA) for pain reduction [16].

Pain is also a great problem for spastic limb rehabilitation in subjects with upper motor neuron syndrome. The same origin and mechanisms seen for CD have been postulated for pain due to stroke or traumatic brain injury, so BoNT-A can be used for reducing pain and spasticity. The possibilities of increasing the passive range of motion (p-ROM) of the upper spastic limb, reducing spasticity, and improving posture and pain during p-ROM assessment, measured using the Numeric Rating Scale (NRS), have been hypothesized by Trompetto et al., in a retrospective study in 70 post-stroke patients treated with BoNT-A for upper limb hypertonia [17].

At the end of the study, the authors postulated that pain reduction after BoNT-A treatment can be induced via the muscle relaxant effect acting on spastic dystonia and/or via a specific action along the nociceptive pathway acting on the sensory neurons, thus permitting a passive range of motion increase in stroke survivors.

Finally, we know the reasons why many patients with spasticity want to be treated with BoNT-A; however, we do not know why many subjects have discontinued treatment. Cinone et al. evaluated the reasons for and determinants of BoNT-A discontinuation in patients with stroke, multiple sclerosis, spinal cord injury, and traumatic brain injury by conducting a retrospective study of 56 discontinuer patients treated with botulinum toxin for up to ten years.

The main reasons for discontinuation were logistic problems (due to distance or the absence of an adequate caregiver) and surgical interventions for spasticity, including intrathecal baclofen.

The authors concluded that it is crucial to identify the possible predictors of discontinuation to improve the efficacy of multidisciplinary management while also confirming the crucial roles of rehabilitation and caregivers in achieving better long-term outcomes [18].
